# Early-Season Detection of Influenza A(H3N2) Subclade K in Wastewater, Colorado, USA, 2025–2026

**DOI:** 10.3201/eid3213.260431

**Published:** 2026-08

**Authors:** Molly C. Hetherington-Rauth, Van Nguyen, Emily Washeleski, Kevin Castro Vital, Gabrielle Lequia, Deborah Aragon, Nisha B. Alden, Kirsten Weisbeck, Laura Bankers, Alexandria Rossheim, Allison Wheeler, Shannon R. Matzinger

**Affiliations:** Colorado Department of Public Health and Environment, Denver, Colorado, USA

**Keywords:** influenza, influenza virus, wastewater surveillance, viruses, influenza A, H3N2, next-generation sequencing, genomics, deconvolution, United States

## Abstract

We assessed the value of wastewater sequencing for monitoring influenza circulation in Colorado, USA, focusing on the emergence of influenza A(H3N2) subclade K. We detected subclade K in wastewater 31 days before clinical detection. Our results demonstrated that wastewater sequencing can provide real-time situational awareness for public health officials.

Seasonal influenza A and B viruses cause significant illness and death annually (*1*,*2*). During the early 2025–26 influenza season, an antigenetically drifted influenza A(H3N2) strain, known as subclade K, emerged with reduced antibody binding to contemporaneous vaccine strains (*3*,*4*). The emergence of subclade K was correlated with early seasonal influenza activity observed in several countries (*5*). Colorado was among 3 states in the United States to observe early influenza activity based on outpatient respiratory illness reported to ILINet (*6*)*.* By the week ending November 22, 2025, Colorado reported high activity.

The Colorado Department of Public Health and Environment supplemented conventional surveillance, including hospitalization rates, clinical specimen sequencing, and wastewater detection using digital PCR (dPCR), with wastewater sequencing for genetic surveillance. We sequenced the influenza A virus hemagglutinin (HA) gene from wastewater and assessed its added value for monitoring influenza trends during the respiratory illness season.

## The Study

### Hospitalization Cases

**Figure 1:**
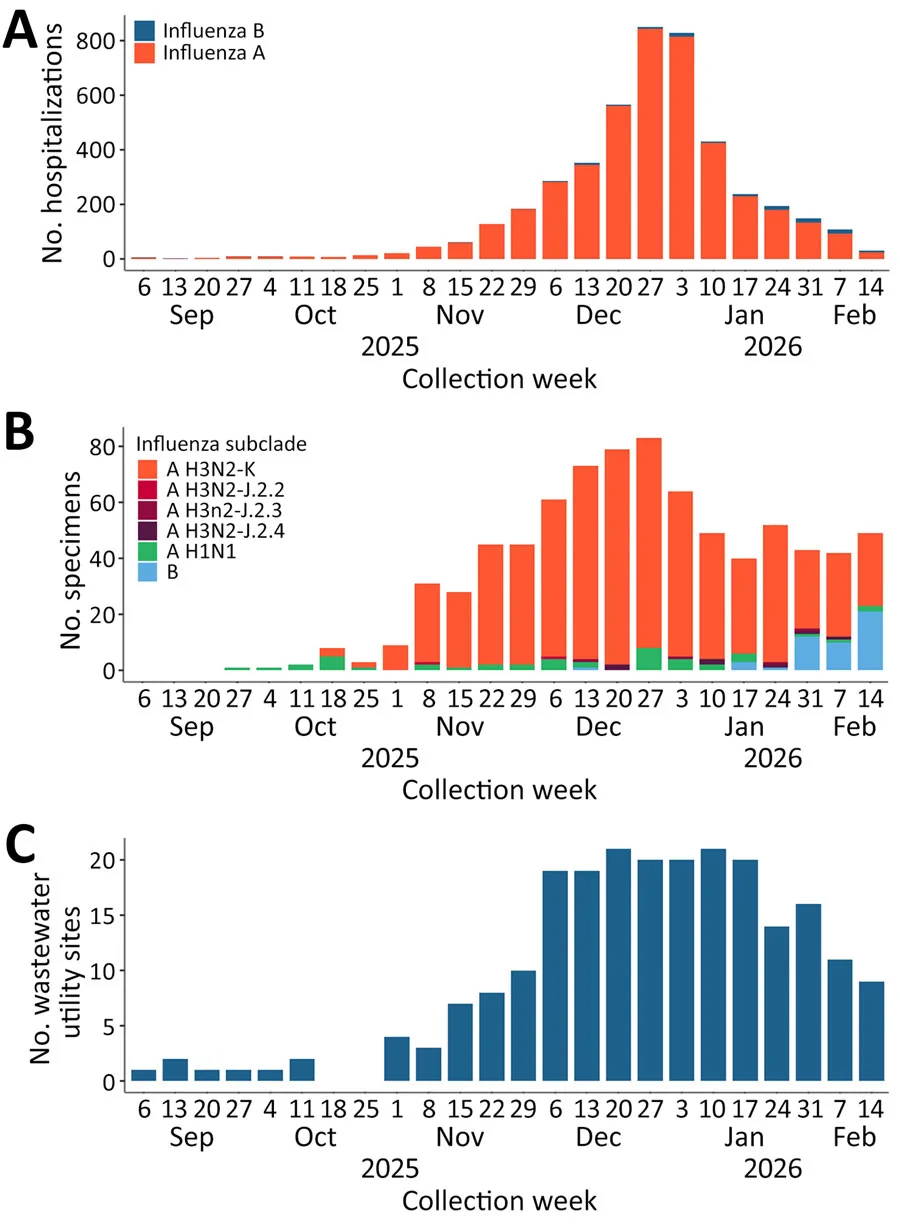
Comparison of hospitalizations, clinical specimens sequenced, and wastewater detection in study of early-season detection of influenza A(H3N2) subclade K in wastewater, Colorado, USA, 2025–2026. A) Influenza hospitalizations of influenza A and influenza B case-patients, by collection week. B) Clinical specimens sequenced and grouped by subclade characterization, by collection week. C) Unique wastewater utility sites at which concentrations of influenza A were above the limit of quantification, by collection week. No sites had concentrations above the limit of quantification for the weeks ending October 18 and 25, 2025. Collection week is shown by the week ending date.

Colorado requires the reporting of influenza-associated hospitalizations. We retrieved data for influenza-associated hospitalizations with admission dates of September 1, 2025–February 14, 2026, from the statewide surveillance system. During that period, Colorado recorded 4,426 influenza A–associated hospitalizations and 110 influenza B–associated hospitalizations. Cases began increasing the week ending November 25, 2025, and peaked the week ending December 27, 2025 (Figure 1, panel A).

### Clinical Sequencing

The state laboratory received up to 5 influenza A–positive clinical specimens weekly from hospitals of both inpatient and outpatient cases participating in the statewide influenza sentinel surveillance system. We subtyped specimens and sequenced those meeting sequencing criteria (Appendix); we sequenced 812 specimens during September 1, 2025–February 14, 2026. Of those specimens, 720 (89%) were influenza A(H3N2). Of those samples, we characterized 704 (98%) as influenza A(H3N2) subclade K (hereafter, subclade K) (Figure 1, panel B). The first sample characterized as subclade K was collected on October 12, 2025. Colorado’s right-size target is 50 samples per week to confidently provide situational awareness; we reached that threshold the week ending December 6, 2025. 

### Wastewater Detection

Established in 2020, the Colorado Wastewater Surveillance Program monitors wastewater across the state for respiratory viruses and emerging pathogens using dPCR (*7*). The state laboratory received samples 2×/week from 21 sentinel wastewater treatment facilities. We retrieved wastewater detection data for September 1, 2025–February 14, 2026. We detected and quantified influenza A, influenza A H1, and influenza A H3 by using previously described methods (M.C. Hetherington-Rauth et al., unpub. data, https://doi.org/10.1101/2025.10.15.25338105) (Appendix). 

We defined the limit of detection by dPCR as the presence of 1 positive partition. We refer to the limit of quantification by dPCR as 1,200 gene copies (gc)/L. Concentration values calculated that fall below the limit of quantification can be interpreted to mean that target genetic material was present but cannot be accurately quantifiable. Concentration values recorded as 0 /L indicate no positive partitions were observed. The number of sewersheds with influenza A viral concentration above our limit of quantification began increasing the week ending November 8, 2025, and peaked the week ending December 20, 2025, mirroring influenza hospitalization trends (Figure 1, panel C). 

**Figure 2:**
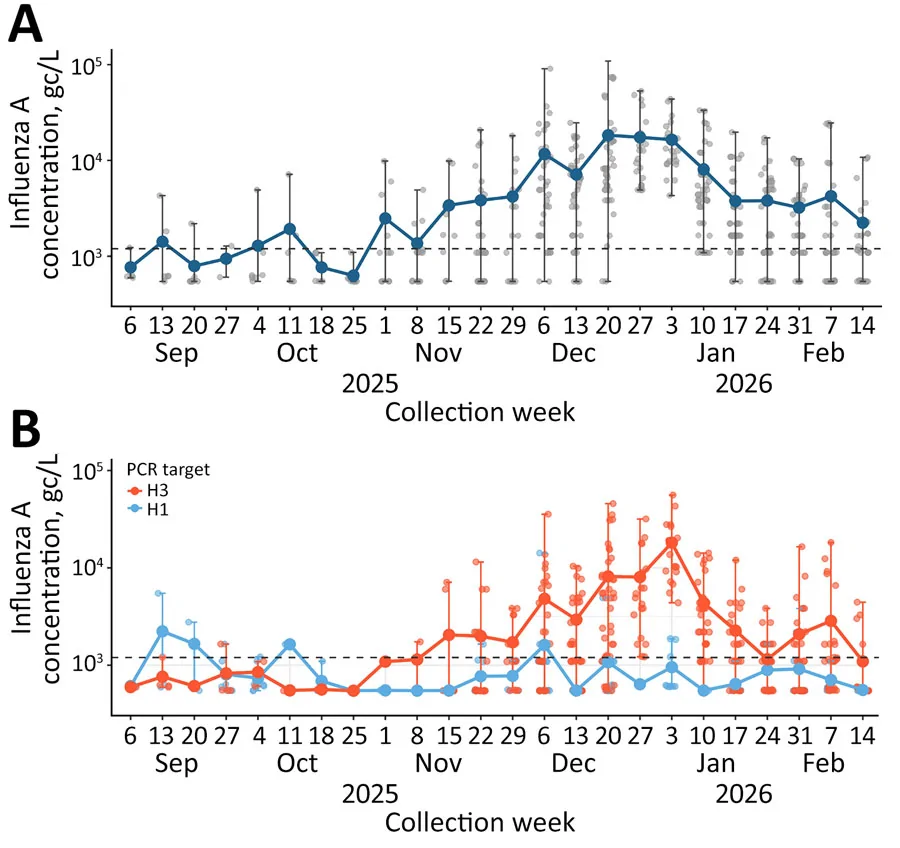
Influenza A viral concentration in wastewater in study of early-season detection of influenza A(H3N2) subclade K, Colorado, USA, 2025–2026. A) Influenza A target concentration. Blue points and connecting lines show the average concentration and trend by week. Gray points show concentrations for each sample collected by week. Error bars show the range of concentration by week. Dashed horizontal line indicates the defined limit of quantification, 1,200 gc/L. No sites had influenza A detection for the weeks ending October 18 and 25, 2025. B) Influenza H1 and H3 concentration in wastewater. Points and connecting lines show the average concentration and trend by week. Error bars show the range of concentration by week. Dashed horizontal line indicates the defined limit of quantification, 1,200 gc/L. No sites had H3 detections for the weeks ending October 4, 11, 18, and 25 and November 1, 2025. There were no H1 detections for the weeks ending October 18 and 25 and November 1, 8, and 15, 2025, or for January 10 and 17 and February 7 and 14, 2026. Concentration is shown in log_10_ scale. Collection week is shown by the week ending date. gc, gene copies.

The average viral concentration of influenza A in wastewater peaked the week ending December 27, 2025 (Figure 2). Influenza A H3 average viral concentrations mirrored the average influenza A viral concentrations. Influenza A H1 average viral concentrations remained low at or below the level of quantification.

### Wastewater Sequencing

**Figure 3:**
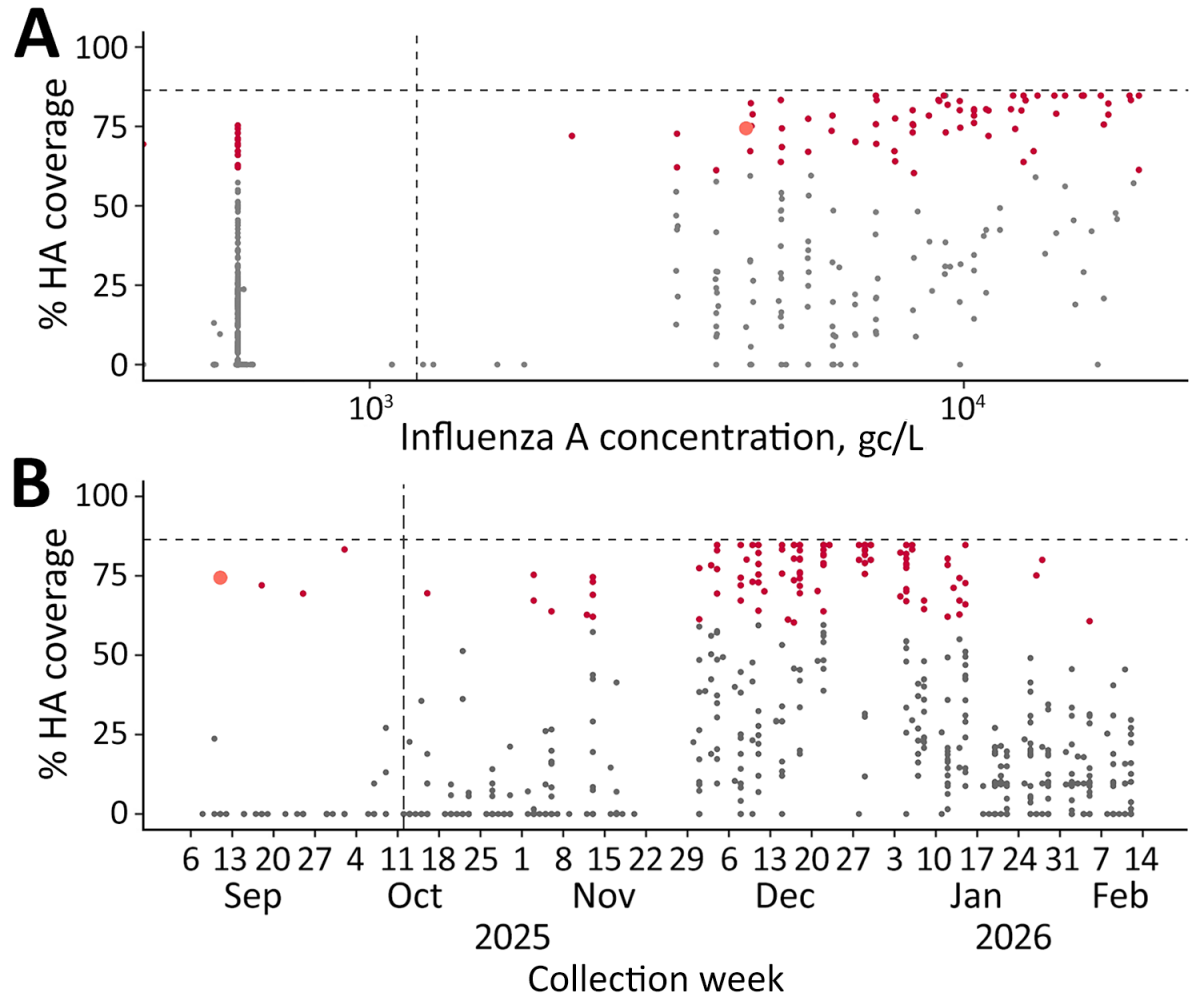
Wastewater sequencing metrics in study of early-season detection of influenza A(H3N2) subclade K in wastewater, Colorado, USA, 2025–2026. A) Percentage coverage of HA gene plotted against influenza A viral concentration. Orange represents sample with the first subclade K detection, collected October 16, 2025. Red represents samples with >60% coverage required for performing deconvolution. Gray represents samples with <60% coverage. Horizontal dashed line indicates the tiled amplicon scheme, which covers 86.7% of the HA gene reference sequence. Vertical dashed line indicates the defined limit of quantification, 1,200 gc/L. B) Percentage coverage of HA gene plotted against sample collection date. Horizontal dashed line indicates the tiled amplicon scheme, which covers 86.7% of the HA gene reference sequence. Vertical dashed line indicates the collection date of the first clinical sample characterized as subclade K, October 23, 2025. gc, gene copies; HA, hemagglutinin.

The state laboratory sequenced the HA gene from wastewater samples using previously described methods (M.C. Hetherington-Rauth et al., unpub. data, https://doi.org/10.1101/2025.10.15.25338105) (Appendix). We sequenced 649 samples collected during September 1, 2025–February 14, 2026. Of the samples sequenced, 112 (17%) met the 60% coverage threshold of the HA gene required for deconvolution (subclade characterization and abundance estimation) (Appendix). We observed that influenza A viral concentration in wastewater was moderately correlated with percent coverage of the HA gene (0.66 Spearman correlation coefficient) (Figure 3, panel A).

We first identified subclade K in wastewater from a sample collected on September 11, 2025; we detected subclade K by clinical sequencing 31 days later (Figure 3, panel B; Appendix Figure). The sample had 74.4% coverage of the HA gene; subclade K was detected at 100% relative abundance. (Coverage is the percentage of the reference gene sequenced at 10× depth; abundance is the estimated relative frequency of the subclade in the sample.) Viral concentration of influenza A in the sample was 4,300 gc/L. After the initial detection, we observed subclade K at near 100% abundance in nearly all wastewater samples meeting the 60% coverage threshold. We detected subclade J.2.4 in 2 samples at 11% and 10% abundance and subclade J.2.3 in 3 samples at 83%, 64%, and 12% abundance. We observed subclade J.2 and J.2.1 at <1% abundance on average. We detected subclade K in 20 of 21 sentinel sites. We detected subclade K in 13 wastewater samples with influenza A viral concentrations below the limit of quantification.

## Conclusions

We assessed the value of wastewater sequencing alongside hospitalization data, clinical sequencing, and wastewater viral quantification to track influenza trends during the respiratory illness season. During the early 2025–26 influenza season, wastewater viral concentration trends mirrored hospitalization trends. The characterization of wastewater and clinical samples revealed that the rise in cases correlated with the emergence of subclade K. Using wastewater sequencing, we detected subclade K 31 days earlier than in clinical samples. Furthermore, the detection of subclade K in wastewater samples with influenza concentrations below the limit of quantification by dPCR demonstrates the high sensitivity of wastewater sequencing.

In addition to earlier detection, wastewater sequencing captures asymptomatic and less severe cases, as well as cases from underserved communities and persons who do not seek medical care, compared with conventional genetic surveillance systems that rely on specimens from hospital case-patients (*8*,*9*). Although wastewater samples inherently provide aggregate population-level data, they are subject to factors including the number of infected persons and population mobility, such as tourism and travel between work, home, and school. To clarify how those factors could affect the interpretation of wastewater data, especially for local public health response, future studies could compare clinical and wastewater data at finer geographic scales.

Integrating wastewater sequencing into existing surveillance systems provides an innovative approach to monitor genetic variation, especially early in an influenza season when clinical specimens are typically scarce. Genetic characterization of the virus is integral to monitoring antigenic drift, antiviral resistance markers, vaccine drift, and mutations in primer/probe binding regions of diagnostic assays at national scale. Early characterization of genetic changes of influenza virus through wastewater sequencing can strengthen public health action and reduce response time.

Appendix Additional information about early-season detection of influenza A(H3N2) subclade K in wastewater, Colorado, USA, 2025–2026.
