# Supplementary material for: Early-Season Detection of Influenza A(H3N2) Subclade K in Wastewater, Colorado, USA, 2025–2026
Source: Emerg Infect Dis. 2026 Aug;32(Suppl 1):S66–70. doi: 10.3201/eid3213.260431 (PMC13585669; doi:10.3201/eid3213.260431)
Supplement: Supplementary file 1 [file 26-0431-Techapp-s1.pdf]

Article DOI: https://doi.org/10.3201/eid3213.260431

EID cannot ensure accessibility for supplementary materials supplied by authors. Readers who have difficulty accessing supplementary content should contact the authors for assistance.

Early-Season Detection of Influenza A(H3N2) Subclade K in Wastewater, Colorado, USA, 2025–2026

Appendix

Supplemental Methods

Clinical Sequencing and bioinformatic analysis. The state laboratory received up to 5 influenza A positive clinical specimens weekly from hospitals collected from outpatient and inpatient cases participating in the statewide influenza sentinel surveillance system. We subtyped clinical samples using CDC's Human Influenza Virus Real-Time RT-PCR Diagnostic Panel. We reflexed samples with a Ct value of 28 or lower for sequencing as part of our Influenza Sequencing Center activities. Specimens were sequenced following the CDC's whole genome influenza sequencing protocol (*1*).

Genomic assembly and analysis were performed using the CDPHE-influenza pipeline (*2*). Briefly, we filtered and trimmed reads using SeqyClean (v1.10.09) (*3*). We used IRMA (*4*) for genome assembly of the filtered reads. We characterized consensus sequences to the subclade level using Nextclade (*5*). We retrieved clinical sequencing data from September 1, 2025, to February 14, 2026.

Wastewater detection and quantification. Wastewater samples from Colorado's 21 wastewater sentinel surveillance sites were submitted twice weekly to the State Public Health Laboratory for detection and quantification by digital-PCR (dPCR) using methods described previously (*6*). Briefly, wastewater was concentrated using Nanotrap Microbiome A Particles and Nanotrap Enhancement Reagent 1 (CERES nanoscience) and nucleic acid material was extracted using the Applied Biosystems MagMAX wastewater Ultra DNA/RNA extraction kit (FisherScientific) on the KingFisherFlex system (FisherScientific). Murine Hepatitis Virus (MHV) was used as a recovery control. dPCR was performed using the QIAcuity OneStep Advanced Probe Kit

(Qiagen) and a set of custom primer and probes targeting the M gene for influenza A detection and quantification, and the HA gene for influenza A subtypes H1 and H3 detection and quantification. The initial concentration of the target gene was calculated as

$$Viral\ Concentration\ (copies/uL) * \frac{Reaction\ Volume}{Template\ Volume} * \left(\frac{1}{Extraction\ Factor * Additional\ Concentration}\right).$$

Wastewater sequencings and bioinformatic analysis. Wastewater samples collected between September 1, 2025 and February 14, 2026 with at least one positive partition by dPCR for either the influenza A (M gene target), H1 (HA gene target) or H3 (HA gene target) were sequenced.

We sequenced the HA gene segment for the H3 subtype using a tiled amplicon based sequencing protocol with a custom tiled amplicon primer scheme covering 86.7% of the HA gene. Methods have been previously described (*7*). Briefly, we used the SuperScript III One-Step RT-PCR System with Platinum Taq High for cDNA synthesis and amplification. Sequencing libraries were prepared using the Illumina DNA Prep Kit. Final library pools were sequenced on either the Illumina Nextseq2000 or Illumina MiSeq i100 platforms to generate 2x150 bp reads.

Assembly of the HA gene was performed using the CDPHE-H5-influenza pipeline (*7*). We filtered reads for quality using fastp v0.32.2 (*8*). Filtered reads were aligned to the A/Wisconsin/67/2025 (H3N2) (GenBank accession no. CY163680.1) HA gene sequence using bwa v0.7.17-r1188 (*9*). Primer sequences were trimmed using samtools amplicon clip v1.21 (*10*). Resulting bam files were used for subclade characterization and abundance estimation, also called deconvolution. Deconvolution was performed on samples with at least 60% coverage using Freyja (*11*) with influenza A H3 barcodes (version 2025–12–09), which included subclade K references.

References

1. Zhou B, Donnelly ME, Scholes DT, St. George K, Hatta M, Kawaoka Y, et al. Single-reaction genomic amplification accelerates sequencing and vaccine production for classical and Swine origin human influenza A viruses. J Virol. 2009;83:10309–13. PubMed https://doi.org/10.1128/JVI.01109-09

2. CDPHE-influenza. https://github.com/CDPHE-bioinformatics/CDPHE-influenza/tree/v1.1.2

3. Zhbannikov IY, Hunter SS, Foster JA, Settles ML. SeqyClean: a pipeline for high-throughput sequence data preprocessing. In: Proceedings of the 8th ACM International Conference on Bioinformatics, Computational Biology, and Health Informatics. Boston: Association for Computing Machinery; 2017. p. 407–16.

4. Shepard SS, Meno S, Bahl J, Wilson MM, Barnes J, Neuhaus E. Viral deep sequencing needs an adaptive approach: IRMA, the iterative refinement meta-assembler. BMC Genomics. 2016;17:708. PubMed https://doi.org/10.1186/s12864-016-3030-6

5. Aksamentov I, Roemer C, Hodcroft E, Neher R. Nextclade: clade assignment, mutation calling and quality control for viral genomes. J Open Source Softw. 2021;6:3773. https://doi.org/10.21105/joss.03773

6. Hetherington-Rauth MC, Nguyen V, Lequia G, Pysnack N, Bankers LA, Rossheim AEB, et al. Targeted tiled amplicon based protocol for sequencing the Hemagglutinin (HA) gene segment of seasonal influenza A and influenza B virus from wastewater at high depth of coverage. Public and Global Health; 2025 [cited 2026 Feb 23]. https://doi.org/10.1101/2025.10.15.25338105

7. CDPHE-H5-influenza. https://github.com/CDPHE-bioinformatics/CDPHE-H5-influenza/tree/v1.0.0-edge

8. Chen S, Zhou Y, Chen Y, Gu J. fastp: an ultra-fast all-in-one FASTQ preprocessor. Bioinformatics. 2018;34:i884–90. PubMed https://doi.org/10.1093/bioinformatics/bty560

9. Li H. Aligning sequence reads, clone sequences and assembly contigs with BWA-MEM. 2013 [cited 2026 May 1]. https://arxiv.org/abs/1303.3997 doi:10.48550/ARXIV.1303.3997

10. Li H, Handsaker B, Wysoker A, Fennell T, Ruan J, Homer N, et al.; 1000 Genome Project Data Processing Subgroup. The Sequence Alignment/Map format and SAMtools. Bioinformatics. 2009;25:2078–9. PubMed https://doi.org/10.1093/bioinformatics/btp352

11. Karthikeyan S, Levy JI, De Hoff P, Humphrey G, Birmingham A, Jepsen K, et al. Wastewater sequencing reveals early cryptic SARS-CoV-2 variant transmission. Nature. 2022;609:101–8. PubMed https://doi.org/10.1038/s41586-022-05049-6

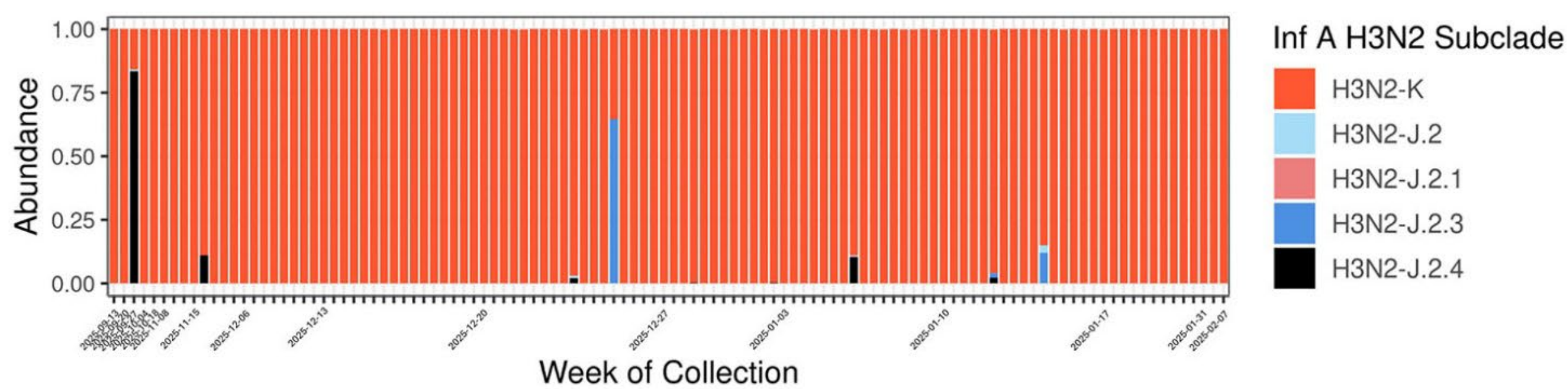

Appendix Figure. Influenza A H3N2 subclade abundance in wastewater samples in study of early-season detection of influenza A(H3N2) subclade K in wastewater, Colorado, USA, 2025–2026. Each bar represents an individual wastewater sample that received >60% coverage across the HA gene. Bars are ordered and grouped by collection week; the horizontal axis does not represent a consistent time scale.
